# Low protein-induced intrauterine growth restriction as a risk factor for schizophrenia phenotype in a rat model: assessing the role of oxidative stress and neuroinflammation interaction

**DOI:** 10.1038/s41398-023-02322-8

**Published:** 2023-02-01

**Authors:** Larissa Allgäuer, Jan-Harry Cabungcal, Catherine Yzydorczyk, Kim Quang Do, Daniella Dwir

**Affiliations:** 1 Center for Psychiatric Neuroscience, Department of Psychiatry, Lausanne University Hospital (CHUV) and University of Lausanne (UNIL), Lausanne, Switzerland; 2 Department Woman-Mother-Child, Division of pediatrics, DOHaD Laboratory, Lausanne University Hospital (CHUV) and University of Lausanne (UNIL), Lausanne, Switzerland

**Keywords:** Molecular neuroscience, Schizophrenia

## Abstract

A large body of evidence suggests that intrauterine growth restriction (IUGR) impedes normal neurodevelopment and predisposes the offspring to cognitive and behavioral deficits later in life. A significantly higher risk rate for schizophrenia (SZ) has been reported in individuals born after IUGR. Oxidative stress and neuroinflammation are both involved in the pathophysiology of SZ, particularly affecting the structural and functional integrity of parvalbumin interneurons (PVI) and their perineuronal nets (PNN). These anomalies have been tightly linked to impaired cognition, as observed in SZ. However, these pathways remain unexplored in models of IUGR. New research has proposed the activation of the MMP9-RAGE pathway to be a cause of persisting damage to PVIs. We hypothesize that IUGR, caused by a maternal protein deficiency during gestation, will induce oxidative stress and neuroinflammation. The activation of these pathways during neurodevelopment may affect the maturation of PVIs and PNNs, leading to long-term consequences in adolescent rats, in analogy to SZ patients. The level of oxidative stress and microglia activation were significantly increased in adolescent IUGR rats at postnatal day (P)35 as compared to control rats. PVI and PNN were decreased in P35 IUGR rats when compared to the control rats. MMP9 protein level and RAGE shedding were also increased, suggesting the involvement of this mechanism in the interaction between oxidative stress and neuroinflammation. We propose that maternal diet is an important factor for proper neurodevelopment of the inhibitory circuitry, and is likely to play a crucial role in determining normal cognition later in life, thus making it a pertinent model for SZ.

## Introduction

Schizophrenia (SZ) is a complex neurodevelopmental disease, which etiopathology involves the interaction between genetic and environmental risk factors [1–5]. This interaction during specific, sensitive periods of brain development underlies the vast heterogeneity of symptoms, among which cognitive deficits are the earliest to appear [6–8]. Adverse environmental events that have been described to increase the risk for SZ are maternal infections, obstetrical complications, maternal stress, and nutritional deficiencies during the perinatal period [7, 9–14]. More specifically, evidence has shown that low caloric intakes during pregnancy increase the lifetime risk for SZ [1], as was observed in individuals conceived during famines that occurred in the Netherlands (1944–1945) and China (1959–1961) [9, 15, 16]. Indeed, recent studies have highlighted the potential role of intrauterine growth restriction (IUGR) in the pathophysiology of psychiatric neurodevelopment diseases, such as SZ [17–19]. IUGR [20] is widely considered to be a predisposing factor to several non-transmissible diseases such as cardiovascular and renal disorders [21–23]. In addition to significantly impacting birth weight [20, 24], IUGR has also been correlated with impaired cerebral development [20, 25, 26] and adverse cognitive outcomes, such as lower IQ and reduced working memory [24, 27], occurring even many years after the adverse environmental exposure. Interestingly, these features are also found in SZ patients at the early stages of the disease, suggesting a role of IUGR in the pathophysiology of SZ. To understand the mechanisms by which IUGR may lead to brain impairments related to SZ, IUGR was modeled in animals as gestational caloric restriction or isocaloric protein restriction [28–31]. More recently, IUGR induced by moderate under-nutrition has been demonstrated to cause mild cerebral impairments such as delayed cortical synaptogenesis and myelination, increased oxidative damage, and inhibitory circuitry impairments [30]. Despite this recent study, little is known about the mechanism induced by IUGR that could lead to changes in inhibitory brain circuitry during development.

Interestingly, oxidative stress, neuroinflammation [9, 32–38] as well as impairment of the structural and functional integrity of parvalbumin-expressing fast-spiking interneurons (PVI) [39] and their surrounding perineuronal net (PNN) [40–43] were established as hallmarks of SZ pathophysiology [32, 44–46]. PVI are particularly sensitive to oxidative stress and neuroinflammation during the early postnatal period [47–50], supporting a major role of environmental stressors occurring during a well-defined period of brain development [10, 51, 52]. The interaction between oxidative stress and neuroinflammation during the early postnatal stage was shown to impair PV/PNN maturation in an animal model of redox dysregulation with a SZ-like phenotype [47, 53]. This interaction was mediated by the activation of the matrix metalloproteinase 9 (MMP9) which induces the shedding of the receptor for advanced glycation end-product (RAGE), leading to a feedforward loop of oxidative stress and neuroinflammation [53], deleterious for PVI maturation. Therefore, the MMP9/RAGE mechanism may be a good candidate for the induction of PVI impairments and further brain development deficits, underlying the adverse cognitive outcomes induced by IUGR.

The focus of this study is to assess whether a maternal low protein diet exposes the offspring to a higher risk of oxidative stress and microglia activation, leading to a deleterious impact on the neurodevelopment of PVIs and their PNNs.

## Material and methods

### Animal model

IUGR in pregnant Sprague Dawley rats (240–294 g, Charles River, L’Arbresle, France) were prepared as previously described [29, 31] (Suppl Fig. 1). These experiments were approved by the ethics committee for animal research at the University of Lausanne, Switzerland. Rats had free access to water and were kept at standard laboratory conditions with controlled temperature and humidity, under a 12-h light/dark cycle. Briefly, pregnant dams belonging to the control group are exposed to a diet composed of 23% casein (representing a normal isocaloric diet; SAFE U8959, version 1, Augy, France), while dams from the IUGR group are fed with an isocaloric diet composed of 9% casein (low protein; SAFE U8959, version 40). Both diets begin at gestational day 1 (G1), defined as the day on which sperm was detected in the vaginal smear, and maintained ad libitum until birth. A normal diet was administrated at birth and until the date of sacrifice, on postnatal day (P35). The control group consisted of 5 rats and the IUGR group of 7. Pregnant dams were assigned randomly to the control or the IUGR group. Only males were used for this study, which was approved by the Swiss cantonal veterinary office.

Rats were anesthetized at P35 for intracardial perfusion with filtered 4% paraformaldehyde in PBS, pH 7.4 solution, enabling brain fixation. Brains were dissected and post-fixed for storage in 30% sucrose. 50 µm coronal sections of the rat brains were prepared with a microtome (Microm HM440E) for immunohistochemistry procedure. Coronal sections were stored in ethylene-glycol for preservation at −20 °C.

### Immunofluorescence

For each rat (*n* = 5–7), brain sections (3–4 sections per rat) containing our region of interest (the anterior cingulate cortex) were used for IH quantification of proteins. The following antibodies were incubated at 4 °C for 48 h: anti-Iba1 (1:1000; Abcam, ab5076), anti-CD68 (1:1500; Abcam, ab53444), anti-8-oxoDG (8-Oxo-2’-deoxyguanosine) (1:350; Trevigen, 4354-MC-050), anti-PV (1:50,000; Swant, PV 25), anti-SST (1:100; Millipore; MAB354), anti-WFA (Wisteria Floribunda Lectin) for PNN staining (1:50,000; Sigma, L1516), anti-extracellular RAGE (1:300; MAB1179, R&D systems), anti-intracellular RAGE (1:500; ab3611, Abcam), and anti-MMP9 (1:500; Santa Cruz, sc-10737). Sections were then incubated with fluorescent secondary antibody: goat anti-mouse (1:300; A488; Life Technologies, USA), goat anti-rabbit IgG (1:300; CY3; Chemicon International, USA), streptavidin 405 conjugate (1:300; A405; Millipore Corporation, USA), and chicken anti-goat Alexa 594 (1:300, A494: Abcam, UK). For the staining of membrane-bound RAGE and intranuclear RAGE, IH was performed as previously described [53]. Briefly, to identify RAGE shedding, an antibody targeting the extracellular domain of RAGE was used together with another antibody targeting the intracellular domain of RAGE, both revealed with two different secondary antibodies.

### Confocal imaging and image analysis

Sections were visualized and processed with a Zeiss confocal microscope (LSM 710 Quasar) controlled by the Zen software (Carl Zeiss AG, Switzerland). Z stacks of 12 images (1.87 µm interval) were scanned (1024 × 1024 pixels, ×20 or ×40 objectives) for analysis with IMARIS 6.4.0 (Bitplane AG, Switzerland). The region of interest (ROI) was marked throughout the Z-stacks ACC images in which the number of PV-immunoreactive (IR) cells, PNN (WFA-labeled PNNs), and SST-expressing neurons were quantified. 8-oxo-dG and Iba-1 labeling intensities were obtained using the Coloc module to calculate 8-oxo-dG and Iba-1 fluorescent intensity (in arbitrary unit: a.u). For RAGE and MMP9 staining, ×40 objective was used, covering the ACC area with 4 images. To obtain the number of PVIs and PVIs surrounded by PNNs (WFA + PVI) the spots module which assigns spot markings to the profile-labeled voxels of a given size (~9 and ~5 µm, respectively) was used. Spots generated for PVI profiles (>9 µm) that touched/overlapped with spots generated for PNN ( > 5 µm) were considered as PVIs surrounded by PNNs (WFA-positive PV). To separately obtain the Iba-1 cell count, spots generated for Iba-1 profiles in the range of 3–5 µm were considered as Iba-1 positive cells.

### Statistical analysis

The analyses were done blindly, as different persons were in charge of the animal processing and the immunofluorescence analyses (immunofluorescence processing, confocal scanning and Imaris analyses), and number ID were given to the animals.

The sample size was chosen to detect approximately 25% change in the number of PV-IR cells and approximately 75% change in 8-oxo-dG intensity with a power of 80% at a significant α-value set to p = 0.05. To ensure that the sample size used had adequate power, data was checked using the tool power details in JMP v12.2. The threshold for conservative power was always taken above 0.7. Type 1 error was set at 0.05 (alpha) and the difference to detect between means was taken at the level of 0.7–0.8. Raw data and residuals of the model were checked for normal distribution (with JMP goodness-of-fit and R software) using Shapiro-Wilk Test (with acceptance value probability of *p* > 0.05) and variance homogeneity was evaluated using Bartlett test Test (with acceptance value probability of *p* > 0.05). For some of the variables (PV intensity, PNN count, 8-oxo-DG intensity, Iba1 intensity, RAGE ratio), the homogeneity criteria was not met, so the data were converted using logarithmic transformation, which homogenized their variance. Upon detection of a significant main effect in multivariate ANOVA, the mean number of PVI, PNN cells, PVI with PNN (PNN + PVI), MMP9, RAGE, and the overall 8-oxo-dG, Iba-1 intensity/count and CD68 were compared between IUGR and control groups using Dunnett’s method.

## Results

### Intrauterine protein deficiency does not lead to persistent low body weight

Rats were weighed before being anesthetized at P35 (adolescence). Bodyweight at P35 showed no significant difference between IUGR (132 g ± 9.6) and control (145 g ± 15.3) groups (Table 1). This shows that low-protein induced IUGR during gestation does not define a persistent low postnatal body weight at P35, suggesting that the phenotype described in our study is not related to growth retardation per se.

**Table 1:** Comparison of weight, PV intensity, PV + cell count, WFA intensity and WFA + cell count, PV + WFA + cell count, SST + cell count, 8-oxo-dG intensity, Iba1 intensity, Iba1+ cell count, CD68 + cell count, MMP9 + cell count and RAGE ratio (Intra-RAGE/Extra-RAGE) in the anterior cingulate cortex (ACC) between control and IUGR rats at P35.

	Body weight	PV intensity	PV count	WFA intensity	WFA cell count	PV + PNN count	8-oxo-dG intensity	Iba1 intensity	Iba1 count	CD68 count	SST count	MMP9 count	RAGE ratio
Control (*n* = 5)	145 ± 15.3	5.2 ± 3.38	67.88 ± 5.24	3.55 ± 3.07	49.69 ± 7.21	42.06 ± 6.83	0.32 ± 0.14	2.24 ± 1.79	97.69 ± 75.01	49.2 ± 35.48	4.01 ± 1.10	177.9 ± 49.83	3.97 ± 0.73
IUGR (*n* = 7)	132 ± 9.6	1.93 ± 1.34	40.26 ± 7.29	0.61 ± 0.55	26.7 ± 5.90	21.17 ± 5.21	1.24 ± 0.57	3.64 ± 1.20	166.55 ± 84.93	134.85 ± 64.32	2.62 ± 1.59	283.94 ± 84.12	12.15 ± 3.72
*p*-value		<0.01**	<0.0001****	<0.01**	< 0.0001****	<0.0001****	<0.0001****	<0.01**	<0.01**	<0.05*	<0.01**	<0.05*	<0.001***

Results are mean ± STD, *p < 0.05, **p < 0.01, ***p < 0.001, ****p < 0.0001 Control vs IUGR, *n* = 5–7.

### Intrauterine protein deficiency leads to increased oxidative stress and microglial activation in the ACC

**Fig. 1:**
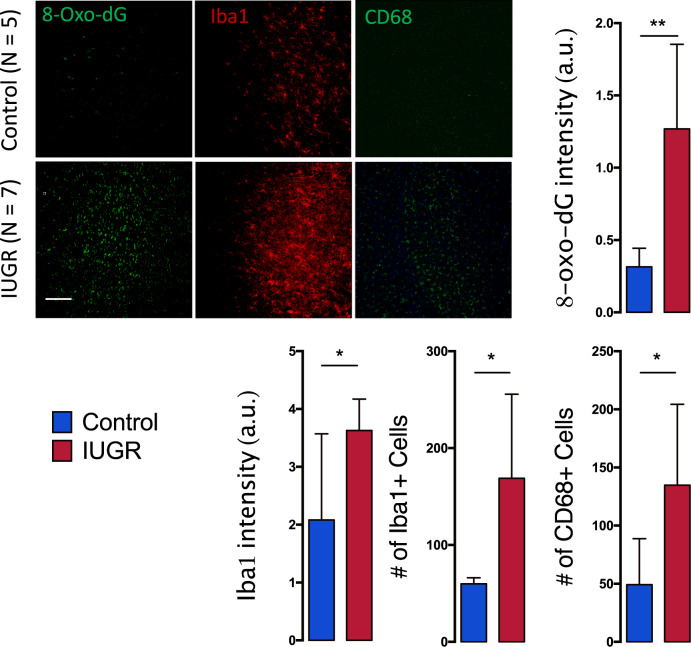
Increased oxidative stress and microglia activation in the low protein induced IUGR offspring at P35. Images for 8-oxo-dG (label for mitochondrial DNA oxidation), Iba-1 (label for microglia) and CD68 (label for activated microglia) in both control (*n* = 5) and IUGR (*n* = 7) groups. IUGR leads to significant increase in 8-oxo-dG intensity (a.u.), Iba-1 intensity (a.u.) and Iba-1 cell count, as well as CD68 cell count in the ACC as compared to controls. Results are mean ± STD, *p = 0.05, **p < 0.01, ****p < 0.0001 Control vs IUGR. Scale bar: 60 μm.

We first investigated whether an intra-uterine low protein diet was sufficient to induce oxidative stress in the anterior cingulate cortex (ACC) of P35 offspring. 8-oxo-dG, which marks DNA oxidation, was used to detect the extent of oxidative stress damage [54]. The ACC of P35 IUGR rats showed significantly higher 8-oxo-dG intensity as compared to control rats (*p* < 0.0001; Fig. 1), suggesting increased oxidative stress induced by the intrauterine protein deficiency in IUGR rats.

As oxidative stress is tightly linked to neuroinflammation [35], we further explored microglia activation, by analyzing Iba1 intensity and number of cells. Iba-1 immunolabeling revealed an increase of Iba-1-positive cells in the ACC of IUGR rats compared with control rats (*p* = 0.01) as shown in Fig. 1. Likewise, Iba-1 expression in ACC of IUGR rats, as shown by Iba-1 intensity (a.u), exhibited a significant increase compared to control rats (*p* = 0.01). Increased microglia activation was further corroborated by an increase in CD68 labeling (*p* = 0.03) (Fig. 1). These findings indicate that intrauterine protein deficiency-induced IUGR increases the number of activated microglia as well as the protein expression of Iba-1 and CD68 in the ACC of P35 rats.

Altogether, a maternal low protein diet leads to increased oxidative stress and an upregulation of the total number of microglial cells, increasing neuroinflammation, in the ACC of individuals born after IUGR.

### Intrauterine protein deficiency impairs PVIs and PNNs maturation in the ACC

**Fig. 2:**
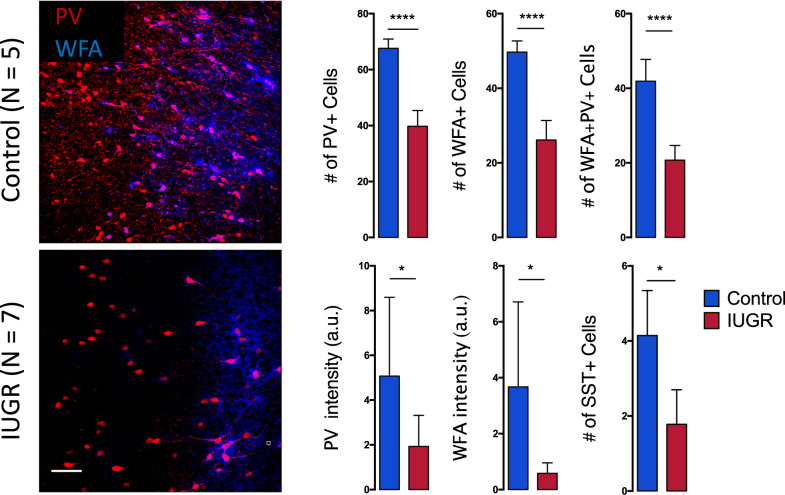
Decreased PV, PNN, and SST in the low protein induced IUGR offspring at P35. Images for PV, WFA (label for PNN) and WFA-positive PVI in both control (*n* = 5) and IUGR (*n* = 7) groups. IUGR leads to significant decrease in PV + cells, PV intensity (a.u.), number of WFA + cells, WFA intensity (a.u.), number of WFA + PV + cells, and SST + cells in the ACC as compared to controls. Results are mean ± STD, *p = 0.05, **p < 0.01, ****p < 0.0001 Control vs IUGR. Scale bar: 60 μm.

To investigate whether the observed oxidative damage and microglia activation in the ACC of intrauterine protein deficiency-induced IUGR rats affects the inhibitory circuitry, we analyzed PV and PNN expressing neurons. We found that the immunolabeling intensity of PV but also the number of PV-IR cells were lower (PV count: *p* < 0.0001 and PV intensity: *p* < 0.05) in the ACC of IUGR rats compared with those of control rats (Fig. 2). This suggests a predominant decrease in the number of PVI, more than the amount of PV protein that they express. Interestingly, somatostatin (SST)-IR cells were also decreased in the IUGR rats, suggesting other impairments in the inhibitory circuitry induced by the intrauterine protein deficiency (Fig. 2).

Furthermore, intrauterine protein deficiency significantly impaired the extracellular matrix component as revealed by WFA (a lectin that recognizes PNN around PVI) intensity (*p* < 0.01) and WFA-IR (PNN) cell count (*p* < 0.001) as compared to control rats. Double-immunofluorescent labeling for WFA-positive PVI showed a significantly reduced number of WFA-IR PV-IR cells (*p* < 0.0001) in the ACC of P35 IUGR rats when compared to control rats (Fig. 2).

### MMP9/RAGE mechanism is induced in the ACC by intrauterine protein deficiency

**Fig. 3:**
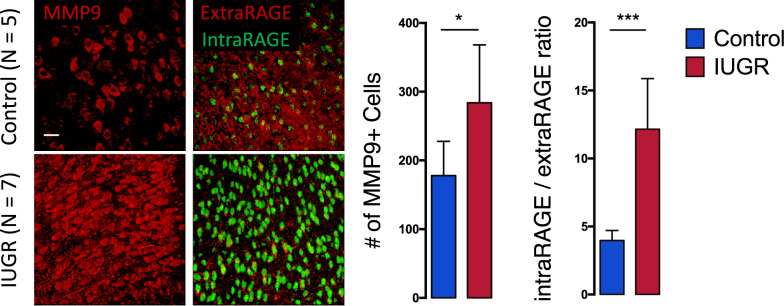
Increased MMP9 and RAGE shedding in the low protein induced IUGR offspring at P35. Images for MMP9, and double immunostaining for intranuclear RAGE positive cells (green) and membrane-bound RAGE positive cells (red), in both control (*n* = 5) and IUGR (*n* = 7) groups. IUGR leads to significant increase in MMP9 + cells and RAGE ratio (number of intranuclear RAGE positive cells/membrane-bound RAGE positive cells) in the ACC as compared to controls. Results are mean ± STD, *p = 0.05, **p < 0.01, ****p < 0.0001 Control vs IUGR. Scale bar: 30 μm.

Environmental insults inducing oxidative stress and neuroinflammation may affect the maturation of PV/PNN. As previously observed, oxidative stress activates MMP9 and RAGE shedding, which together leads to a feedforward loop of oxidative stress and neuroinflammation, impairing PVI maturation [53]. We investigated whether this mechanism may be induced by IUGR and affect PVI/PNN. MMP9-IR cell number was increased in the IUGR rats (*p* < 0.05) as compared to controls (Fig. 3). RAGE shedding, revealed by the ratio of intra-nuclear RAGE staining over the membrane-bound RAGE [53], was also increased in the IUGR rats (*p* < 0.001), indicating increased RAGE shedding induced by MMP9.

### Correlation of oxidative stress/microglia activation markers with PVI deficits

**Fig. 4:**
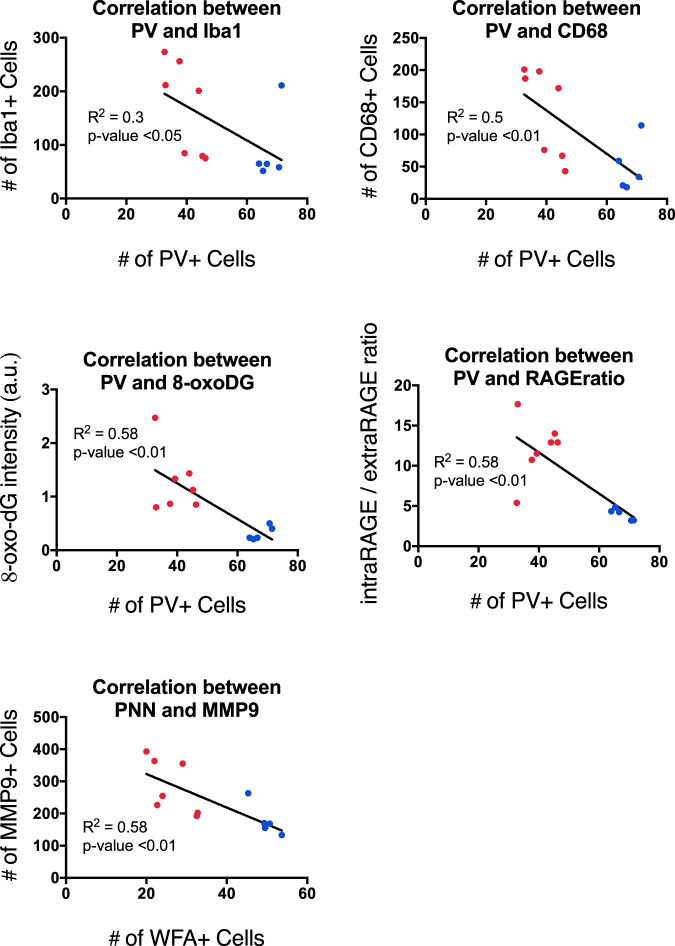
Negative correlation between PV and markers of oxidative stress/microglia activation, as well as between PNN and MMP9 at P35. Negative correlation between PV + cells and Iba1 cell count in IUGR (red) and control (blue) group together (*N* = 12; *R*^2^ = 0.3, *p* < 0.05). Negative correlation between PV + cells and CD68 cell count in IUGR and control group together (*N* = 12; *R*^2^ = 0.5, *p* < 0.01). Negative correlation between PV + cells and 8-oxo-dG intensity in IUGR and control group together (*N* = 12; *R*^2^ = 0.58, *p* < 0.01). Negative correlation between PV + cells and RAGE ratio in IUGR and control group together (*N* = 12; *R*^2^ = 0.58, *p* < 0.01). Negative correlation between WFA + cells and MMP9 + cells in IUGR and control group together (*N* = 12; *R*^2^ = 0.58, *p* < 0.01). *p = 0.05, **p < 0.01.

In order to estimate a link between the oxidative stress and/or microglia activation with the PVI maturation impairments, Iba1, CD68 and 8-oxo-DG were correlated with PV-IR cells count. Interestingly, these different markers were all negatively correlated with PV, as increased oxidative stress and microglia activation were associated with decreased PV (Fig. 4). Moreover, MMP9 was negatively correlated with PNN, in line with a potential role of this MMP in PNN degradation (Fig. 4). Intriguingly, PV decrease was also associated with increased RAGE shedding, suggesting a role of this process in PVI impairments (Fig. 4).

## Discussion

Overall, our results show that IUGR induced by a low protein diet leads to increased oxidative stress and microglial activation, MMP9/RAGE mechanism activation, leading to the impairment of PVI and PNN maturation at P35. To our knowledge, no studies to date have pinpointed the pathophysiological mechanism linking prenatal malnutrition to subsequent PVI and PNN impairment. Moreover, our study brings a novel insight into the effect of IUGR on brain development through the role of oxidative stress and microglia activation.

Previous studies have already underlined the impact of maternal nutrition on the mental and physical health of the offspring [20, 24, 55, 56]. Offspring exposed to IUGR show altered neurodevelopment, specifically a delay in synaptogenesis [56]. Lack of nutrients during the critical phase of cerebral development provides a hypothesis for the delay in synaptogenesis. Amino acids, the constituents of protein, are the building blocks of neurons, glial cells, and several neurotransmitters, which are essential for physiological neurodevelopment [20]. Furthermore, an intrauterine low protein diet has been shown to have negative consequences on a multitude of physiological neurological functions, such as an altered morphology of the prefrontal cortex, diminished working memory, and reversal learning [1, 18, 19, 26, 27]. However, the specific neural network and regions affected remain unidentified, as for the underlying mechanisms.

### Protein deficiency effect on body weight

We established that there was no significant difference in the protein deficiency-induced IUGR rat’s body weight at P35 compared with that of control rats (Table 1). Whilst not having initial body weights at birth for comparison, previous studies on animal models of gestational low protein diet have shown it to result in low body weight at birth [20, 31, 57]. Based on these previous findings in rat models, we can assume that in our study a significant difference in body weight at birth was later compensated in IUGR rats due to their ad libitum access to an isocaloric diet during the postnatal period. The differences we observed in the ACC of the IUGR P35 rats (Table 1) are therefore not likely to be associated with low body weight at birth, but rather with low protein-associated deficiencies, which occurred during the developmental/gestational period. This suggests that body weight recovery by itself is not sufficient to exclude long-lasting effects caused by an important adverse environmental exposure during gestation (IUGR).

### Increased oxidative stress and microglia activation in IUGR

The research presented here attempts to propose new mechanisms to explain the long-lasting adverse effects due to IUGR. Indeed, we propose that part of the deleterious effects of IUGR are mediated by oxidative stress and microglia activation occurring during fetal brain development. Previous animal models of maternal nutrient restriction have demonstrated increased 8-oxo-dG but also 4-HNE, a marker of lipid peroxidation, indicating increased oxidative damage in offspring born after IUGR [30, 56]. In a model of protein deficiency, increased superoxide production via the NADPH oxidase was observed [20, 31], linking the protein availability to ROS generation. Furthermore, low-protein diet-induced IUGR also leads to neuroinflammatory-associated brain injury [26, 58], presumably via uncontrolled microglia increase [20, 57, 59]. Other animal models of IUGR through uterine artery ligation [24, 60] or placenta embolization [24] and chronic hypoxia [24] also showed increased neuroinflammation, as increased microglia and astrocyte activation.

The results gathered through experimental animal models are supported by observations made in pregnant women. In a translational application of this thought, pregnant women with growth-restricted fetuses revealed a measurable increase in plasmatic oxidants [61], a decrease in antioxidant capacities [20, 62], as well as the presence of a pro-inflammatory environment [17], with increased inflammatory cytokines in individuals born after IUGR [63], changes which are similarly observed in IUGR neonates [20, 26, 58]. Findings of increased biomarkers of oxidative stress in cord blood and placental tissue of infants born after IUGR [56] as well as the induction of a pro-inflammatory environment [17, 64, 65] suggest the long-term effect of the impaired environment.

As for animal models, the cause for growth-restricted foetuses may come from malnutrition of the mother during pregnancy, leading to placenta insufficiency. In humans, placental insufficiency is one of the primary causes of IUGR and has multiple origins, including inappropriate substrate availability due to maternal under- or over-nutrition [26]. A maternal low protein diet induces placental insufficiency and secondary IUGR [19]. One hypothesis linking growth restriction to cognitive deficiency is hypoxia caused by placental insufficiency that affects the neurodevelopment despite brain sparing, a compensation mechanism seen in IUGR in which the blood flow to the brain is increased in an attempt to preserve neurodevelopment [27]. Indeed, although few studies link IUGR to oxidative stress and neuroinflammation in the fetus’ later life, there have been studies showing that IUGR causes placental insufficiency which in turn induces chronic fetal hypoxia [24]. Chronic fetal hypoxia has been linked to cytokine imbalances and redox dysregulation [63].

Fetal growth restriction (FGR) animal models have contributed to establishing the effect of hypoxia, subsequent oxidative stress, and inflammation, on neurodevelopment [24]. Alterations following chronic fetal hypoxia are predominant in region-specific zones leading to deficits in neuronal organization and axonal injury [24]. Moreover, various other diseases such as severe preeclampsia are intricately linked to placental insufficiency [24]. Faulty placentation during early gestation may result in preeclampsia. The fetus’ response to oxygen and nutrient restriction has shown to result in a maternal immune response associated with a systemic low-grade pro-inflammatory state [18]. This, in turn, triggers oxidative stress and inflammation in the fetus (e.g., maternal produced IL-6, a pro-inflammatory cytokine that can traverse the placenta into the fetal circulation), which is associated with impaired neurodevelopment and even specific diseases such as autism spectrum disorder and other developmental delays [18].

### PVI/PNN impairment and IUGR

IUGR seems to be associated with numerous cerebral abnormalities and in particular, brain connectivity impairment [24]. Although such alterations of neurodevelopment are now widely accepted, mechanisms that might explain the associations are still lacking, as data from IUGR in humans remains scarce.

Neuroinflammatory processes, such as cytokines and microglial activation, but also the redox balance, such as antioxidants and oxidative stress, are highly involved in brain development and maturation during gestation [66, 67]. Given the important role of microglia and oxidative stress during development [68, 69], their impairments may lead to long-lasting anomalies in brain structure maturation. Moreover, should these abnormalities caused by IUGR be maintained into the postnatal period and adolescence, they could be a reason for deleterious neurodevelopment and persistent damage to PVIs and PNNs. Indeed, previous studies have shown a lack of antioxidant capacities (such as glutathione) [35, 53, 54, 70] and the presence of pro-inflammatory cytokines [13], such as IL-6 [9, 18, 71] to be deleterious to neurodevelopment (Suppl Table 1). Based on our present observations and previous studies from our lab [47, 53], we further conclude that redox dysregulation and microglia activation induced by FGR affects the normal maturation of PVIs as well as the proper development of their protective PNNs (Figs. 2 and 3). PVI are involved in higher-order sensory and cognitive information processing and their proper maturation, occurring during the early postnatal period, was shown to be detrimental to cognition [72–76]. Indeed, PVI play a major role in the synchronization of pyramidal neurons, inducing a rhythmical firing at gamma frequencies [77], which were shown to be linked to these higher-order processes in the brain [78, 79]. Increased oscillations at gamma frequency were associated with cognitive performance in control subjects [80] but not in SZ patients [81], suggesting a major role of this neural activity in cognition, with impairments in SZ. Although data links IUGR to a higher risk of neurocognitive disabilities, inattention or behavioral disorders, and lower educational levels [20, 24, 27], the ultimate impact of IUGR requires further rigorous investigation. Whilst the lack of evidence linking low FGR with cognition and neurobehavioral tests is a limitation in this study, the correlation between oxidative stress and microglia activation during development and their impact on PVI circuitry, physiology, and function has been well documented [24, 47, 54, 82, 83]. Interestingly, the interaction between oxidative stress and neuroinflammation was previously found to be mediated by MMP9/RAGE mechanism in an animal model with relevance for SZ [53]. Here, the same mechanism was found to be activated, which may lead to the maintenance of the IUGR mediated oxidative stress and microglia activation until the postnatal period and affect PVI maturation [53]. Of note, MMP9 was found to be increased in growth-restricted preterm newborns during postnatal periods [64], in line with our findings. Further investigation will be needed to understand the role of the MMP9/RAGE mechanism in IUGR-induced oxidative stress and microglia activation.

Studies have shown neurocognitive disabilities as well as behavioral disorders during childhood and adolescence in individuals born after IUGR [20, 24, 57]. These behavioral alterations may resemble, without being equivalent to, SZ symptoms, and therefore strengthen the link between IUGR and neurodevelopmental psychiatric disorders. A maternal low protein diet seemingly impacts proper neurodevelopment through an increase in oxidative stress and neuroinflammation and thus hinders what is considered to be normal behavior [43, 47, 56, 84], which may be relevant for SZ. Many adverse events during gestation, such as infection or traumatic events [32, 85, 86], were shown to be risk factors for SZ, and malnutrition, due to famine episodes [9], were associated with a higher risk of developing SZ in the offspring [9, 15, 16]. Therefore, the hypothesis of IUGR inducing increased oxidative stress and neuroinflammation which affect the proper maturation of PVI/PNN may be relevant for SZ.

While redox dysregulation, microglia activation, PVI and PNN impairments have previously each been individually linked to other developmental models, to our knowledge, there are no studies that have highlighted all of these abnormalities in any IUGR model. Moreover, a potential role of the MMP9/RAGE mechanism has never been studied in this model. By evaluating all of the elements mentioned above, our data provides further understanding into how the convergence of environmental risk factors during a specific time of development could potentially contribute to PVI and PNN dysfunction and altered cognition via redox dysregulation and microglia activation.

A limitation of this study includes the lack of the pup’s birth weight, which would have allowed us to be certain of an initial discrepancy between the two groups at birth. An additional lack of follow-up after P35 prevents us from establishing a possible persistence of the neurodevelopmental abnormalities in the ACC of IUGR rats. Indeed, it may be of interest to investigate the long-term impact of an in utero low protein diet on PVI maturation in adulthood, and not only at P35. Behavioral studies of adults could also add insight into the long-term functional impact of IUGR. Studies have demonstrated that PVI and their PNN evolve throughout an individuals’ lifespan, rendering the results of early insults more severe [47, 54]. Finally, a bigger sample size would be needed to reduce interindividual variability and allow a better comparison of the two groups.

In conclusion, this study emphasizes the deleterious impact of maternal protein deficiency during gestation on PVI and PNN circuitry, even if this deficiency is transient and followed by an unlimited normal diet after birth. A temporary deleterious environment during the prenatal period interferes with neurodevelopment even after the individual is no longer exposed to the harmful context. Further research into the impact of a protein-deficient maternal diet during the prenatal period on neuronal synchronization and ulterior behavior and cognition requires attention, and will most certainly strengthen the pathophysiological hypothesis. Studying other mechanisms in IUGR models will allow a better understanding of their intricate links to oxidative stress and neuroinflammation and will offer new therapeutic possibilities targeting different symptoms observed in SZ. This would also pave the way to a window of opportunity for intervention in patients conceived under IUGR, as more than 22 million new-borns are affected by low birth weight every year [26]. Through a better understanding of the impact of a low protein IUGR on neurodevelopment and the mechanism leading to the deficits observed, this study hopes to add insight into the possible phenotype of SZ in patients born to pregnant women with a protein insufficiency dietary lifestyle.

## Supplementary information

Supplementary Material

Supplementary Figure 1
