# Supplementary material for: Low protein-induced intrauterine growth restriction as a risk factor for schizophrenia phenotype in a rat model: assessing the role of oxidative stress and neuroinflammation interaction
Source: Transl Psychiatry. 2023 Feb 1;13:30. doi: 10.1038/s41398-023-02322-8 (PMC9889339; doi:10.1038/s41398-023-02322-8)
Supplement: Supplementary file 1 — Supplementary Material [file 41398_2023_2322_MOESM1_ESM.docx]

| **Animal models for IUGR** | **Oxidative stress** | **Inflammation** |
| --- | --- | --- |
| Maternal protein deficiency (Armengaud et al., 2021; Honorio de Melo Martimimiano et al., 2017) | Vascular superoxide anion production by NADPH oxidase (Armengaud et al., 2021) | Increased metabolites of the kynurenin pathway (Honorio de Melo Martimiano et al., 2017) |
| Maternal nutrient restriction (1,2) (Van de Looij et al., 2019 ; Miller et al., 2016) | Increased levels of 8-oxo-dg (marker for DNA oxidative damage) (Van de Looij et al., 2019; Maki et al., 2019)  Increased brain levels of 4-hydroxyneonenal (4HNE) (maker for oxidative stress) (Maki et al., 2019) |  |
| Uterine artery ligation (Miller et al., 2016; Yzydorczyk et al., 2017) |  | Higher levels of OX42 (a marker for microglial density) (Van de Looij et al., 2019) |
| Placental embolization (Miller et al., 2016) |  | Increase of S100B (a biomarker for astrogliosis) (Miller et al., 2016) |
| Chronic hypoxia (Miller et al., 2016) |  | Increased number of activated microglia (Miller et al., 2016) |
| Maternal protein restriction during lactation |  | Increased levels of TNF-α in the offspring (Silva et al., 2010) |
| **IUGR in patients** |  |  |
| Preeclampsia (Armengaud et al., 2021) | Increased placental levels of 8-oxo-dG, 4-hydroxynonenal (4HNE) and redox-factor-1 (ref-1) (Takagi et al., 2004) | Increased number of activated microglia (Miller et al., 2016) (1)  Increase of S100B (a biomarker for astrogliosis) (Miller et al., 2016)  Increased maternal and neonatal IL-6 (pro-inflammatory cytokine) levels (Walker et al., 2015) |
| Intrauterine hypoxia (Lai et al., 2017) |  | Increased pro-inflammatory cytokines in early post-natal days (Armengaud et al., 2021): IL-1β and TNF-α (Lai et al., 2017) |
| Small for gestational age (unknown cause) (Yzydorczyk et al., 2017) | Malondialdehyde (35) and oxidized LDL (Yzydorczyk et al., 2017) | Increased MMP-2 and MMP-9 levels (metalloproteinase) (Yzydorczyk et al., 2017) |
| Schizophrenia patients (Unknown data in IUGR models) | Decreased levels of glutathione (Koga et al., 2016)  Upregulation of superoxide dismutase (SOD) ,  Increased levels of MDA (malionaldehyde) – a product of lipid oxidation/cell membrane damage (Bouvier et al., 2017; Koga et al., 2016; Pizzino et al., 2017) | Downregulation of NRF2 (nuclear factor erythroid 2-related factor 2) (Bouvier et al., 2017; Hardingham et al., 2016)  Increased NADPH (NOX) activity (Do et al., 2009) |

**Supplementary Table 1:** An overview of the various animal models for IUGR and IUGR causes seen in patients and their respective oxidative stress and inflammatory markers.

**Supplementary Figure 1:** Low protein IUGR induction model in rats. G: gestational age, P: postnatal day, P35: adolescence, P90: early adulthood, arrow: rat lifespan up to P35, end of the experiment.
