# Supplementary figures and images for: Low protein-induced intrauterine growth restriction as a risk factor for schizophrenia phenotype in a rat model: assessing the role of oxidative stress and neuroinflammation interaction
Source: Transl Psychiatry. 2023 Feb 1;13:30. doi: 10.1038/s41398-023-02322-8 (PMC9889339; doi:10.1038/s41398-023-02322-8)

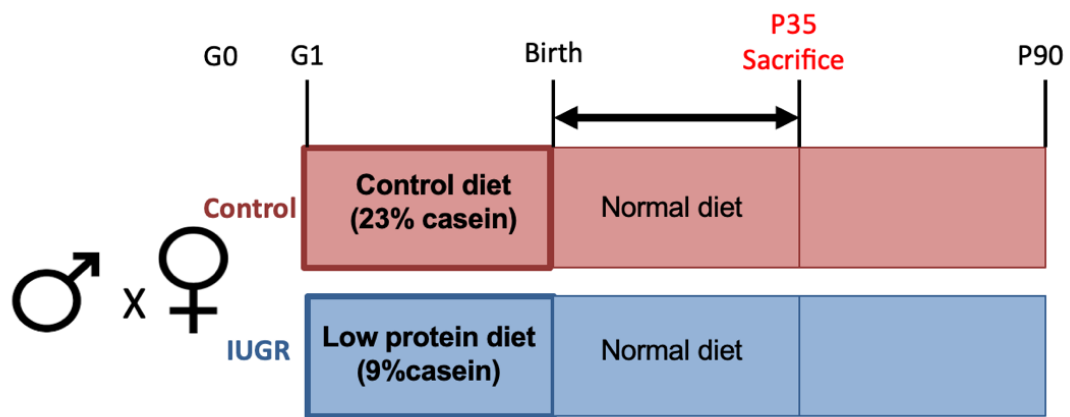

Supplement: Supplementary file 2 — Supplementary Figure 1 [file 41398_2023_2322_MOESM2_ESM.pdf]
